# Genetic variation at *ERBB3*/*IKZF4* and sexual dimorphism in epitope spreading in single autoantibody-positive relatives

**DOI:** 10.1007/s00125-021-05546-9

**Published:** 2021-08-26

**Authors:** Julie Vandewalle, Bart J. Van der Auwera, Henna Amin, Erik Quartier, Aster K. Desouter, Sylvie Tenoutasse, Pieter Gillard, Christophe De Block, Bart Keymeulen, Frans K. Gorus, Mark Van de Casteele, the Belgian Diabetes Registry

**Affiliations:** 1 Diabetes Research Center, Vrije Universiteit Brussel (VUB), Brussels, Belgium; 2 Diabetes Clinic, Universitair Ziekenhuis Brussel (UZ Brussel), Brussels, Belgium; 3 Diabetology Clinic, Hôpital Universitaire des Enfants Reine Fabiola (HUDERF), Université Libre De Bruxelles, Brussels, Belgium; 4 Department of Endocrinology, University Hospitals Leuven, Leuven, Belgium; 5 Department of Endocrinology, Diabetology and Metabolism, Universitair Ziekenhuis Antwerpen, Edegem, Belgium

**Keywords:** Beta cell function, *ERBB3*, Gender, *IKZF4*, Prediabetes, Prediction, Sex, SNP, Type 1 diabetes

## Abstract

**Aims/hypothesis:**

We examined whether the non-HLA susceptibility locus *ERBB3/IKZF4* influences progression of type 1 diabetes stage specifically according to sex.

**Methods:**

SNPs of *ERBB3* (rs2292239 T/G) and *IKZF4* (rs1701704 G/T) were screened by allelic discrimination quantitative PCR assay in first-degree relatives of type 1 diabetes patients who had developed at least one circulating autoantibody. The effect of *ERBB3/IKZF4* genotypes and sex, on the progression of single autoantibody positivity to multiple autoantibody positivity and from multiple autoantibody positivity to diabetes, was studied by Kaplan–Meier analysis and multivariate Cox regression.

**Results:**

In the cohort of autoantibody-positive first-degree relatives, the risk allele frequencies for *ERBB3* rs2292239 (T) and *IKZF4* rs1701704 (G) were increased. There was a significant male excess at the stage of multiple autoantibody positivity (*p* = 0.021). In Kaplan–Meier survival analysis, progression from single to multiple antibody positivity was delayed in female participants with genotype *ERBB3* GG (*p* = 0.018, vs *ERBB3* TG+TT) or *IKZF4* TT (*p* = 0.023, vs *IKZF4* GT+GG), but not in male participants. In multivariate Cox regression models, the interaction effects between female sex and *ERBB3* GG (*p* = 0.012; HR = 0.305 [95% CI 0.120, 0.773]) or between female sex and *IKZF4* TT (*p* = 0.011; HR = 0.329 [95% CI 0.140, 0.777]) emerged as potential determinants of delayed progression to multiple autoantibodies. The progression from multiple autoantibody positivity to type 1 diabetes appeared not to be influenced by *ERBB3/IKZF4*.

**Conclusions/interpretation:**

In siblings and offspring of type 1 diabetes patients, polymorphism in region *ERBB3/IKZF4* may affect disease progression at the level of epitope spreading in female individuals. Our findings suggest that interaction between sex and *ERBB3/IKZF4* may contribute to the post-pubertal male excess in type 1 diabetes.

**Graphical abstract:**

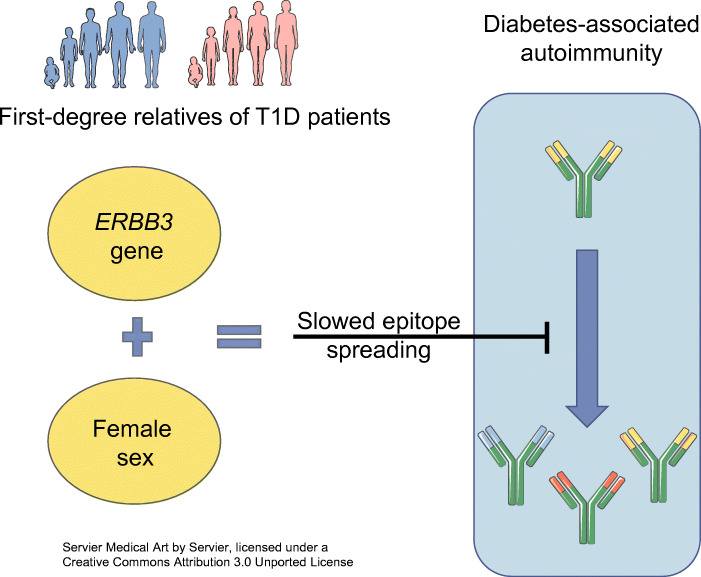

**Supplementary Information:**

The online version of this article (10.1007/s00125-021-05546-9) contains peer-reviewed but unedited supplementary material.

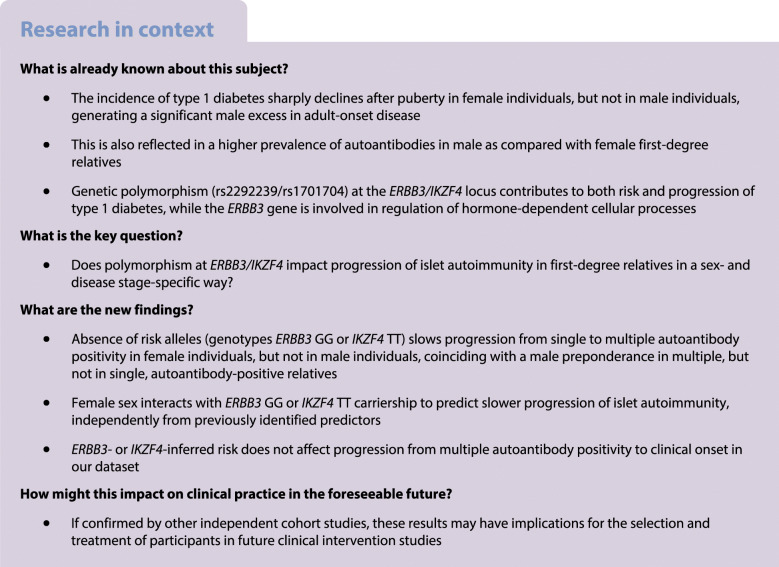



## Introduction

Type 1 diabetes is characterised by an immune-mediated destruction of pancreatic beta cells leading to insulin deficiency. Unlike other autoimmune diseases, it exhibits a strong male bias for diagnosis after age 15 years due to a steep post-pubertal drop in incidence in female individuals only [1, 2]. This is also reflected in a higher prevalence of islet autoantibodies (autoAbs) in male than in female first-degree relatives (FDRs), especially after age 10 years [3]. It has been suggested that this male excess may relate to positive direct and indirect effects of 17betaOH-oestradiol (E2) and oestrogen receptors on beta cell formation, function and survival [4, 5].

In children at genetic or familial risk followed from birth, male sex was reported to confer a higher risk of developing islet autoimmunity by age 6 years [6], but to date there are no indications of more rapid disease progression in autoantibody-positive (autoAb^+^) male individuals. If anything, multiple autoAb^+^ girls were reported to develop clinical onset more rapidly than boys [7]. However, the striking age-dependent disease heterogeneity [8] also warrants investigations in older risk groups which generate the majority of individuals eligible for prevention trials, as well as the majority of new-onset patients [1].

In our cohort of persistently autoAb^+^ FDRs sex was not an independent determinant of progression from single to multiple autoAb positivity, or from multiple positivity to clinical onset, in multivariate analysis [9, 10]. However, non-HLA polymorphisms may exert a stage-specific impact on disease progression in at-risk (sub)groups in time-to-event analysis [11–13]. We wondered whether confirmed non-HLA susceptibility genes encoding proteins expressed in beta cells and implicated in cell survival and proliferation may contribute to sexual dimorphism in progression of subclinical islet autoimmunity. In this context *ERBB3* (which encodes erb-B2 receptor tyrosine kinase 3 [ERBB3]) emerged as a prime candidate to be investigated, as ERBB3 is expressed in various cell types including beta cells [14], and can modulate expression and transcriptional activity of oestrogen receptors [15]. Genetic variation in the *ERBB3/IKZF4* region was repeatedly associated with type 1 diabetes [14] and used to improve prediction of islet autoimmunity and disease progression [11, 13]. We hypothesised that *ERBB3*, or nearby genes, may exert stage-related and/or sex-related effects on the progression of asymptomatic disease in a population at increased familial risk. We selected SNPs rs2292239 and rs1701704, located near *ERBB3/IKZF4*, to investigate this in a cohort of autoAb^+^ FDRs followed by the Belgian Diabetes Registry (BDR) [9, 10].

## Methods

### Participants

**Fig. 1:**
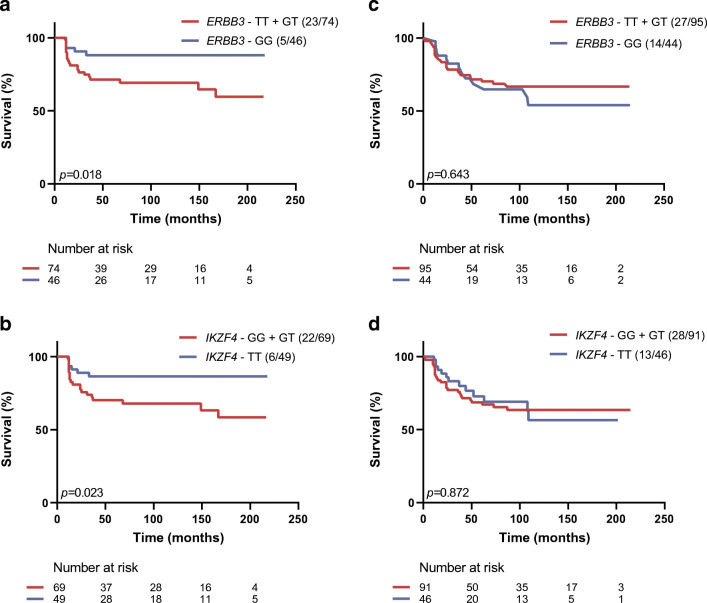
Sex-specific effect of *ERBB3* and *IKZF4* on the development of multiple autoAbs. Kaplan–Meier survival plots for conversion from single to multiple autoAb positivity according to presence (red line) or absence (blue line) of at least one risk allele for *ERBB3*-rs2292239 (**a**, **c**) or *IKZF4*-rs1701704 (**b**, **d**) in either female (**a**, **b**) or male (**c**, **d**) FDRs. For each arm the genotype and number (events/cases) are indicated above the graph. The numbers of individuals at risk are indicated below the *x*-axis. Significant effects (*p*<0.05) were observed for *ERBB3* and *IKZF4* in female participants, but not in male participants

The BDR identified and followed 462 persistently autoAb^+^ siblings and offspring (under 40 years of age at first positivity) of type 1 diabetes patients between March 1989 and December 2015 among a group of 7029 FDRs enrolled after informed consent from the relatives or their legal representative [9, 10]. Progression of the relatives through the different stages of subclinical autoimmunity is visualised in Electronic supplementary material (ESM) Fig. 1 (see ESM Methods: Participants, for further details).

### Analytical methods

AutoAbs against insulin (IAA), GAD65 (GADA), insulinoma-associated antigen-2 (IA-2A) and zinc transporter 8 (ZnT8A) were previously measured by liquid-phase radiobinding assays and *HLA-DQ* and *HLA-A* genotypes by allele-specific oligonucleotide hybridisation [9, 10]. *ERBB3* rs2292239 and *IKZF4* rs1701704 were genotyped by allelic discrimination using TaqMan SNP genotyping assays C_15967467_10 and C_8340619_10, respectively (cat no. 4351379, Applied Biosystems, Foster City, CA, USA), on a QuantStudio 12 K Flex Real-Time PCR System (Applied Biosystems) (see ESM Methods: Analytical methods, for further details).

### Statistical analyses

Statistical differences between groups were analysed with the Pearson χ^2^ test for categorical variables and with the Kruskal–Wallis test for continuous variables. Kaplan–Meier survival analysis with logrank test and multivariate Cox regression analysis were used to assess progression from single to multiple autoAb positivity and from multiple autoAb positivity to diabetes for different SNP genotypes, according to sex. Two-tailed statistical tests were performed and *p* values <0.05 were considered significant (see ESM Methods: Statistical analyses, for further details).

## Results

### *ERBB3* rs2292239 and *IKZF4* rs1701704 genotypes

Both SNPs had call rates >98% within our autoAb^+^ FDR cohort (ESM Fig. 1). The minor and major allelic frequencies of both SNPs differed significantly from those in the European population in the 1000 Genomes Project (*p* < 0.01; ESM Table 1, ESM Methods), with an increased prevalence of the *ERBB3* T and *IKZF4* G risk alleles in the cohort. Genotype distributions for both SNPs did not deviate significantly from the Hardy–Weinberg equilibrium (*p* > 0.05; ESM Table 1). General characteristics of the study population are presented per *ERBB3/IKZF4* genotype and subclinical stage (ESM Tables 2, 3). There was a significant male excess at the stage of multiple autoAb positivity (*p* = 0.021; ESM Table 3), but not at the stage of single autoAb positivity (*p* = 0.38; ESM Table 2).

### *ERBB3/IKZF4* SNPs and progression from single to multiple autoAb positivity

Both SNPs affected epitope spreading according to sex in Kaplan–Meier analysis. Progression from single to multiple autoAb positivity was slowed in female participants without *ERBB3* risk (T) alleles (*p* = 0.018 vs female participants carrying ≥1 T allele; Fig. 1a), and in female participants without *IKZF4* risk (G) alleles (*p* = 0.023 vs presence of ≥1 G allele; Fig. 1b). No such delay was observed in male participants (Fig. 1c,d).

The suggested interaction between female sex and *ERBB3* GG or *IKZF4* TT genotype for delaying epitope spreading was further examined by multivariate Cox regression analysis. Stepwise conditional forward models were built separately for *ERBB3* and *IKZF4* (Table 1). In both models, the absence of IAA as first autoAb (*p* < 0.05), older age at first autoAb positivity (*p* < 0.001), absence of the *HLA-DQ2/DQ8* high-risk genotype (*p* < 0.001) and presence of the HLA-A*24 allele (*p* < 0.02) delayed the development of multiple autoAbs (Table 1), in line with previous findings [9, 10]. In both the model for *ERBB3* and for *IKZF4,* being female or carrying the low-risk genotype (GG and TT, respectively) did not impact progression to multiple autoAb positivity when considering both variables separately (Table 1). However, the interaction effect between *ERBB3* GG and female sex (*p* = 0.012; HR = 0.305 [95% CI 0.120, 0.773]) or between *IKZF4* TT and female sex (*p* = 0.011; HR = 0.329 [95% CI 0.140, 0.777]) significantly delayed epitope spreading (Table 1). No interaction between *ERBB3* or *IKZF4* and the previously reported independent determinants of epitope spreading [10] reached significance in multivariate analysis (Table 1).

**Table 1:** Cox regression analysis of progression from single autoAb positivity to multiple autoAb positivity in FDRs

Variable	Model *ERBB3*		Model *IKZF4*	
	*p*	HR (95% CI)	*p*	HR (95% CI)
Age first autoAb^+^	<0.001	0.913 (0.881, 0.947)	<0.001	0.914 (0.882, 0.947)
Non-IAA (0/1^a^)	0.037	0.576 (0.343, 0.967)	0.043	0.585 (0.348, 0.983)
Sex (0/1^b^)	NM		NM	
HLA-A*24 (0/1^a^)	0.017	0.405 (0.193, 0.851)	0.011	0.381 (0.181, 0.802)
Non-(*HLA*-*DQ2/DQ8*) (0/1^a^)	<0.001	0.332 (0.193, 0.570)	<0.001	0.349 (0.203, 0.600)
*ERBB3*-GG (0/1^a^)	NM		–	
*ERBB3*-GG × age first autoAb^+^	NM		–	
*ERBB3*-GG × non-IAA	NM		–	
*ERBB3*-GG × sex	0.012	0.305 (0.120, 0.773)	–	
*ERBB3*-GG × HLA-A*24	NM		–	
*ERBB3*-GG × non-(*HLA-DQ2/DQ8*)	NM		–	
*IKZF4*-TT (0/1^a^)	–		NM	
*IKZF4*-TT × age first autoAb^+^	–		NM	
*IKZF4*-TT × non-IAA	–		NM	
*IKZF4*-TT × sex	–		0.011	0.329 (0.140, 0.777)
*IKZF4*-TT × HLA-A*24	–		NM	
*IKZF4*-TT × non-(*HLA-DQ2/DQ8*)	–		NM	

Models built by multivariate analysis included either *ERBB3* or *IKZF4*

^a^0/1: no/yes

^b^0/1: male/female

NM, not retained in stepwise conditional forward model (*p*>0.050); –, not used as variable in model construction

### *ERBB3/IKZF4* SNPs and progression from multiple autoAb positivity to clinical onset

Cox regression models built for *ERBB3* or *IKZF4* confirmed previously reported independent risk factors (HLA-A*24, IA-2A^+^ or ZnT8A^+^, younger age) for accelerated progression from multiple autoAb positivity to clinical onset [9, 10]. However, progression was not influenced by *ERBB3* or *IKZF4* genotype, be it alone or in interaction with sex or established predictors (ESM Table 4).

## Discussion

Our main finding is that the GG genotype of rs2292239 in the *ERBB3* gene slows progression of subclinical islet autoimmunity in FDRs, but that this effect is restricted to female individuals and to the phase of epitope spreading. Similar results were obtained for the closely linked TT genotype of rs1701704 located near *IKZF4*. This sexual dimorphism in protective action is independent from already known variables associated with slower epitope spreading (older age, presence of HLA-A*24, absence of IAA and/or *HLA-DQ2/DQ8*) [10]. It appears specific for the *ERBB3/IKZF4* region as it was not observed for established predictors of progression. The increased prevalence of *ERBB3/IKZF4* risk alleles in our autoAb^+^ FDR population further suggests that these alleles contribute to the disease risk. The data are also in agreement with the reported male excess in autoAb^+^ individuals [3] and suggest that *ERBB3/IKZF4* may contribute to the male excess observed in FDRs with multiple autoAbs (ESM Table 3). However, these findings require confirmation in independent cohorts.

To date, most studies in autoAb^+^ individuals have been following young children, often from birth after preselection for HLA class II-inferred risk and/or prior islet cell antibody (ICA) testing [11–13]. Our approach, to include also autoAb^+^ adolescents and young adults, is deemed a strength in the context of the present report because autoAbs can appear at any age while most patients develop clinical symptoms in adulthood with a strong post-pubertal male bias [1, 9].

Oestrogens and oestrogen receptors are known to impact beta cell function and formation, as well as immune cell responses, in a sex-dependent way [4, 5]. Since *ERBB3* has been shown to control oestrogen receptor expression and function [15], one may speculate functional interactions between *ERBB3* and oestrogen receptors in beta and/or immune cells to underlie the sex-specific effect of *ERBB3/IKZF4*. It has previously been reported that *ERBB3/IKZF4* polymorphisms associate with higher risk of developing (multiple) autoAbs, and with accelerated progression to clinical onset after seroconversion to single autoAb positivity [12]. Our results suggest that this reported acceleration may rather be interpreted as a selective delay in epitope spreading in *ERBB3* GG/*IKZF4* TT female individuals. Together with the reported lower proneness to autoAb positivity in female vs male individuals [6], this delay may contribute to the selective post-pubertal drop in incidence in female individuals [1], but requires confirmation in multiple autoAb^+^ individuals identified in the general population. Given the low number of FDRs followed from the stage of first autoAb positivity to clinical onset, the impact of the *ERBB3* genotype on overall time to clinical onset could not be accurately determined in this study.

In conclusion, we report that interaction of *ERBB3/IKZF4* and female sex appears to delay the progression from 1 to ≥2 autoAbs and may possibly contribute to lower disease incidence in female individuals, which needs confirmation in independent cohorts of at-risk individuals.

## Supplementary information

ESM (PDF 803 kb)

## Data Availability

The datasets generated and/or analysed during the present study are not openly available as they were derived from pseudonymised data and samples collected by the Belgian Diabetes Registry, a controlled access repository of sensitive human data. They can be made available upon reasonable request to co-author B. Keymeulen, president of the Belgian Diabetes Registry.

## Abbreviations

autoAb: Autoantibody
autoAb^+^: Autoantibody-positive
BDR: Belgian Diabetes Registry
FDR: First-degree relative
IAA: Insulin autoantibodies
IA-2A: Insulinoma-associated antigen-2 autoantibodies
ZnT8A: Zinc transporter 8 autoantibodies
