# Supplementary material for: Genetic variation at ERBB3/IKZF4 and sexual dimorphism in epitope spreading in single autoantibody-positive relatives
Source: Diabetologia. 2021 Aug 26;64(11):2511–6. doi: 10.1007/s00125-021-05546-9 (PMC8494691; doi:10.1007/s00125-021-05546-9)
Supplement: Supplementary file 1 — (PDF 803 kb) [file 125_2021_5546_MOESM1_ESM.pdf]

## ELECTRONIC SUPPLEMENTARY MATERIAL (ESM)

## ESM METHODS

### Participants

For this study (ethically approved under nr. BUN143201939922 by the institutional review board [IRB] of Universitair Ziekenhuis Brussel [UZB]) we received coded samples and data from the Belgian Diabetes Registry (BDR) Biobank (IRB UZB nr BUN143201524128). Belgian autoAb$^+$ FDRs under age 40 were followed and fully characterized in terms of autoAb markers and HLA-inferred risk by BDR. Blood samples were taken every 6-12 months, stored at -80°C, and previously analyzed for diabetes-associated autoAbs and *HLA-DQ* genotype [1,2]. BDR provided previously stored blood and DNA samples (-80°C) and clinical and biological data to the investigators under coded form. DNA was extracted from whole blood as previously described [2]. Follow-up ended at the time of the last blood sampling or at clinical onset of diabetes and amounted to 72 [35-130] months (median [IQR]). We screened for polymorphism of *ERBB3*/*IKZF4* at SNP rs2292239, located in intron 7 of *ERBB3*, and SNP rs1701704, located 5' to *IKZF4* [3]. Of the 462 persistently autoAb$^+$ relatives, 446 could be genotyped for *ERBB3* rs2292239 (100% call rate) and *IKZF4* rs1701704 (98.9% call rate); 16 could not, due to unavailable DNA.

### Analytical methods

Genotyping qPCR assays were performed according to the manufacturer's instructions using Taqman genotyping Master mix, (cat n°4371353, Applied Biosystems), in a total volume of 10 µL with 20 ng genomic DNA, in microamp fast 96-well plates (cat n° 4346906, Applied Biosystems), for 40 cycles, on a QuantStudio™ 12K Flex Real-Time PCR System, and data retrieved using the QuantStudio™ 12K Flex software (Applied Biosystems). Controls without DNA were included in each run. Genotype calling was performed by the software.

### Statistical analyses

Deviation of the male-to-female ratio from that in the background population was analysed by binomial test. Age-matched male-to-female background ratio was calculated based on data retrieved from the Belgian statistical office [4]. Minor and major allele frequencies for *ERBB3* and *IKZF4* were compared to those in the European population (EUR) of the 1000G project [5] by chi-square test. We did not correct for multiplicity (Bonferroni correction) when performing comparisons between groups, but used multivariate Cox regression to adjust for possible confounders. Multivariate Cox regression analysis of survival time was based on forward stepwise conditional modeling and, apart from SNP genotypes, age and sex, included previously identified independent stage-specific predictors of disease progression in the present cohort [1,2], together with their respective interactions with *ERBB3* and *IKZF4* genotypes. For progression from single to multiple autoAb-positivity the variables SNP

genotype, sex, absence/presence of *HLA-DQ2/DQ8*, IAA, *HLA-A*24*, and age at first autoAb positivity were entered in the analysis. For progression from multiple autoAb positivity to clinical onset, the variables SNP genotype, sex, absence/presence of *HLA-A*24* and IA-2A/ZnT8A, being offspring of a diabetic mother, and age at first multiple autoAb positivity were included. The Vittinghoff criterion (at least 5-10 events per variable included [6]) was respected. SPSS version 26.0 software (IBM, Armonk, NY) and Graphpad Prism version 8 software (GraphPad Software, La Jolla, CA) were used.

## ESM REFERENCES


[1] Gorus FK, Balti EV, Messaaoui A, et al. (2017) Twenty-Year Progression Rate to Clinical Onset According to Autoantibody Profile, Age, and HLA-DQ Genotype in a Registry-Based Group of Children and Adults With a First-Degree Relative With Type 1 Diabetes. Diabetes Care 40(8): 1065-1072. 10.2337/dc16-2228

[2] Balke EM, Balti EV, Van der Auwera B, et al. (2018) Accelerated Progression to Type 1 Diabetes in the Presence of HLA-A*24 and -B*18 Is Restricted to Multiple Islet Autoantibody-Positive Individuals With Distinct HLA-DQ and Autoantibody Risk Profiles. Diabetes Care 41(5): 1076-1083. 10.2337/dc17-2462

[3] Keene KL, Quinlan AR, Hou X, et al. (2012) Evidence for two independent associations with type 1 diabetes at the 12q13 locus. Genes Immun 13(1): 66-70. 10.1038/gene.2011.56

[4] *https://bestat.statbel.fgov.be/bestat/crosstable.xhtml?datasource=65ee413b-3859-4c6f-a847-09b631766fa7*

[5] *http://grch37.ensembl.org/Homo_sapiens/Variation/Population?db=core;v=rs2292239;vdb=variation*
*http://grch37.ensembl.org/Homo_sapiens/Variation/Population?db=core;v=rs1701704;vdb=variation*

[6] Vittinghoff, E. and C.E. McCulloch, *Relaxing the rule of ten events per variable in logistic and Cox regression.* Am J Epidemiol, 2007. **165**(6): p. 710-8.

## ESM TABLES

**ESM Table 1.** Actual and expected genotypic distribution for *ERBB3* (rs2292239) and *IKZF4* (rs1701704). Minor allele frequencies for *ERBB3* and *IKZF4* were 0.399 and 0.385, respectively[a]. Genotypic distributions did not deviate from Hardy-Weinberg (HW) equilibrium according to chi-square test.

| Genotype | | n | Frequency | Expected frequency (HW) | Chi² statistics HW |
|---|---|---|---|---|---|
| ***ERBB3*** | **GG** | 156 | 0.350 | 0.361 | |
| | **TG** | 224 | 0.502 | 0.480 | |
| | **TT** | 66 | 0.148 | 0.159 | $p$=0.61 |
| ***IKZF4*** | **TT** | 167 | 0.379 | 0.379 | |
| | **GT** | 208 | 0.471 | 0.474 | |
| | **GG** | 66 | 0.150 | 0.148 | $p$=0.99 |

[a] Minor allele frequencies for *ERBB3* and *IKZF4* were 0.33 in the European population (EUR) of the 1000G project [4].

**ESM Table 2.** Characteristics of single autoAb-positive relatives at baseline (n=259) overall and of subgroups according to *ERBB3* (rs2292239) and *IKZF4* (rs1701704) genotypes.

| | ***ERBB3*** | | | | ***IKZF4*** | | | |
|---|---|---|---|---|---|---|---|---|
| **Variable** | **Overall** | **GG** | **TG** | **TT** | **Overall** | **TT** | **GT** | **GG** |
| **n** | 259 | 90 (35) | 132 (51) | 37 (14) | 255 | 95 (37) | 122 (48) | 38 (15) |
| **Sex** | | | | | | | | |
| **Male** | 139 (54)[a] | 44 (49) | 73 (55) | 22 (59) | 137 (54) | 46 (48) | 68 (56) | 23 (60) |
| **Female** | 120 (46)[a] | 46 (51) | 59 (45) | 15 (41) | 118 (46) | 49 (52) | 54 (44) | 15 (40) |
| ***HLA-DQ8*** | 123 (48) | 46 (51) | 60 (45) | 17 (46) | 121 (48) | 46 (48) | 56 (46) | 19 (50) |
| ***HLA-DQ2/DQ8*** | 51 (20) | 21 (23) | 24 (18) | 6 (16) | 51 (20) | 19 (20) | 26 (21) | 6 (16) |
| ***HLA-A*24*** | 55 (21) | 17 (19) | 31 (24) | 7 (19) | 55 (22) | 17 (18) | 29 (24) | 9 (24) |
| **Duration follow-up (mo)** | 87 [37-144] | 83 [36-136] | 91 [38-148] | 86 [53-135] | 89 [37-145] | 78 [36-122] | 110 [37-164] | 86 [46-134] |
| **Age first autoAb$^+$ (yrs)** | 13 [7-20] | 13 [7-21] | 13 [7-21] | 14 [6-19] | 13 [7-21] | 13 [8-20] | 13 [7-21] | 14 [6-20] |
| **Age at onset (yrs)** | 21 [14-31] | 20 [12-30] | 21 [14-31] | 23 [13-30] | 21 [14-31] | 19 [13-21] | 23 [15-31] | 23 [12-32] |
| **First autoAb** | | | | | | | | |
| **IAA** | 66 (26) | 25 (28) | 32 (24) | 9 (24) | 66 (26) | 26 (28) | 33 (27) | 7 (18) |
| **GADA** | 171 (66) | 59 (66) | 86 (65) | 26 (70) | 167 (66) | 63 (66) | 77 (63) | 27 (71) |
| **IA-2A** | 16 (6) | 4 (4) | 11 (9) | 1 (3) | 16 (6) | 4 (4) | 9 (7) | 3 (8) |
| **ZnT8A** | 6 (2) | 2 (2) | 3 (2) | 1 (3) | 6 (2) | 2 (2) | 3 (3) | 1 (3) |

Data are n(%) unless indicated otherwise. Age and duration are expressed as median[IQR]. [a]M/F ratio (1.16): $p$=0.38 versus M/F ratio (1.03) in age-matched Belgian population (see ESM Methods) by binomial test.

**ESM Table 3.** Characteristics of multiple autoAb-positive relatives (n=256) overall and of subgroups according to *ERBB3* (rs2292239) and *IKZF4* (rs1701704).

| | *ERBB3* | | | | *IKZF4* | | | |
|---|---|---|---|---|---|---|---|---|
| **Variable** | **Overall** | **GG** | **TG** | **TT** | **Overall** | **TT** | **GT** | **GG** |
| **n** | 256 | 85 (33) | 129 (50) | 42 (17) | 255 | 91 (36) | 123 (48) | 41 (16) |
| **Sex** | | | | | | | | |
| **Male** | 149 (58)[a] | 50 (59) | 73 (57) | 26 (62) | 148 (58) | 50 (55) | 73 (59) | 25 (61) |
| **Female** | 107 (42)[a] | 35 (41) | 56 (43) | 16 (38) | 107 (42) | 41 (45) | 50 (41) | 16 (39) |
| ***HLA-DQ8*** | 174 (68) | 61 (72) | 84 (65) | 29 (69) | 173 (68) | 63 (69) | 80 (65) | 30 (73) |
| ***HLA-DQ2/DQ8*** | 76 (30) | 29 (34) | 37 (29) | 10 (24) | 75 (29) | 27 (30) | 39 (32) | 9 (22) |
| ***HLA-A*24*** | 50 (20) | 14 (17) | 27 (21) | 9 (21) | 50 (20) | 19 (21) | 24 (20) | 7 (17) |
| **Duration follow-up (mo)** | 62 [29-119] | 64 [25-121] | 59 [28-120] | 68 [40-117] | 63 [29-120] | 56 [24-109] | 60 [28-127] | 69 [40-115] |
| **Age first multiple autoAb positivity (yrs)** | 10 [6-17] | 10 [6-17] | 9 [5-14] | 15 [6-20] | 10 [6-17] | 10 [6-17] | 9 [5-15] | 14 [6-20] |
| **Age at onset (yrs)** | 16 [10-23] | 16 [11-25] | 15 [10-21] | 22 [11-27] | 16 [10-23] | 16 [10-24] | 15 [10-22] | 21 [12-26] |

Data are n(%) unless indicated otherwise. Age and duration are expressed as median[IQR]. [a]M/F ratio (1.39): $p$=0.021 versus M/F ratio (1.03) in age-matched Belgian population (see ESM Methods) by binomial test.

**ESM Table 4.** Cox regression analysis of progression from multiple-autoAb positivity to type 1 diabetes in first-degree relatives. Models built by multivariate analysis included either *ERBB3* or *IKZF4*.

| | Model *ERBB3* | | Model *IKZF4* | |
|---|---|---|---|---|
| **Variable** | *p* | HR (95% CI) | *p* | HR (95% CI) |
| **Age[a]** | 0.013 | 0.971 (0.948-0.994) | 0.014 | 0.971 (0.949-0.994) |
| **Sex (0/1[b])** | NM | | NM | |
| **Non-*HLA-A*24* (0/1[c])** | 0.002 | 0.523 (0.349-0.785) | 0.002 | 0.522 (0.348-0.784) |
| **Diabetic mother (0/1[c])** | 0.049 | 0.570 (0.326-0.998) | 0.050 | 0.572 (0.327-1.001) |
| **Non-(IA-2A$^+$ and/or ZnT8A$^+$) [d] (0/1[c])** | <0.001 | 0.413 (0.287-0.595) | <0.001 | 0.416 (0.289-0.598) |
| ***ERBB3*-GG (0/1[b])** | NM | | - | |
| ***ERBB3*-GG x age** | NM | | - | |
| ***ERBB3*-GG x sex** | NM | | - | |
| ***ERBB3*-GG x non-*HLA-A*24*** | NM | | - | |
| ***ERBB3*-GG x non-(IA-2A$^+$ and/or ZnT8A$^+$)** | NM | | - | |
| ***ERBB3*-GG x diabetic mother** | NM | | - | |
| ***IKZF4* - TT (0/1[b])** | - | | NM | |
| ***IKZF4* - TT x age** | - | | NM | |
| ***IKZF4* - TT x sex** | - | | NM | |
| ***IKZF4* - TT x non-*HLA-A*24*** | - | | NM | |
| ***IKZF4* - TT x non-(IA-2A$^+$ and/or ZnT8A$^+$)** | - | | NM | |
| ***IKZF4* - TT x diabetic mother** | - | | NM | |

NM, not retained in the stepwise conditional forward model (p>0.050); -, not used as variable in model construction; HR, hazard ratio; [a]age at first multiple autoAb$^+$ sample; [b]0/1: male/female; [c]0/1: no/yes; [d]absence of high-risk autoAb profile

## ESM FIGURE

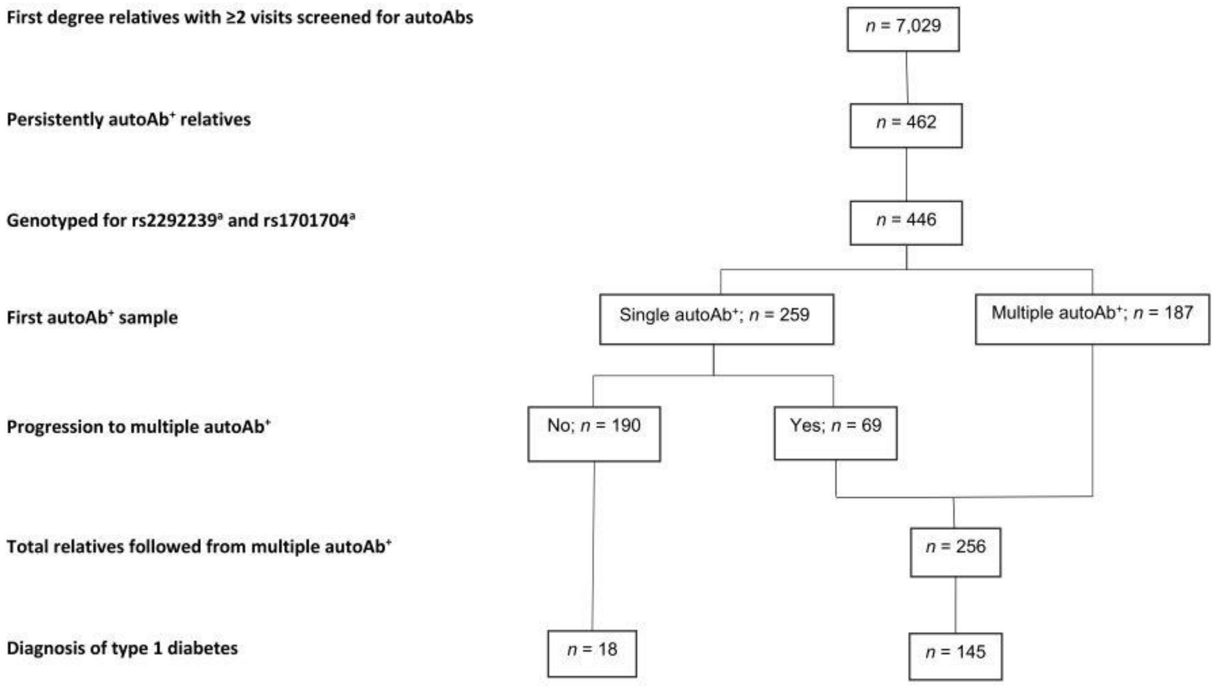


**ESM Fig. 1.** Diagram showing the disposition of FDRs with regards to their progression through the pre-symptomatic stages of type 1 diabetes. [a]*ERBB3* rs2292239 (100% call rate) and *IKZF4* rs1701704 (98.9% call rate).

## ESM Appendix

Members of the Belgian Diabetes Registry who enrolled subjects for this study:

Abrams P, St Augustinus, Wilrijk; Arnouts P, AZ St Jozef, Turnhout; Ballaux D, AZ Nikolaas, Sint-Niklaas; Beckers D, UCL Mt. Godinne, Yvoir; Beckers V, CH St Joseph, Liège; Beirinckx A, AZ St Lucas, Assebroek; Bettens W, AZ KLINA Campus Vesalius, Brasschaat ; Bollaerts K, AZ St Maarten Campus Mechelen, Mechelen; Bosly F, Clinique St Joseph, Arlon; Bouillon R, Legendo, UZ Leuven Gasthuisberg, Leuven; Casteels K, UZ Leuven Gasthuisberg, Leuven; Chivu O, CHR Clinique St Joseph, Liège; Claessens A, Clinique St Joseph, Arlon; Claeys L, AZ St Jozef, Malle; Coeckelberghs M, Kinderziekenhuis Paola, Antwerpen; Coolens J, Jessa Ziekenhuis - Campus Salvator, Hasselt; Coremans P, AZ Nikolaas, Sint-Niklaas; Crenier L, Hôpital Erasme, Bruxelles; Daoudi N, Hôpital Civil Marie Curie, Lodelinsart; Daubresse J, Hôpital Civil Marie Curie, Lodelinsart; De Block C, UZA, Edegem; De Brouckère V, CHU Tivoli, La Louvière; De Feyter I, AZ De Bijloke, Gent; De Schepper J, UZ Brussel, Brussel (Jette); Decochez K, AZ Jan Portaels, Vilvoorde; Decraene P, Imeldaziekenhuis, Bonheiden; Den Brinker M, UZA, Edegem; Derdelinckx L, Clinique Saint Luc, Bouge-Namur; Deweer S, St Elisabeth Ziekenhuis, Zottegem; Dirinck E, UZA, Edegem; Dooms L, Private, Bree; Dotremont H, UZA, Edegem; Driessens S, AZ KLINA, Brasschaat; Duyck F, H Hart Ziekenhuis, Roeselare; Dysseleer A, CH de l'Ardenne, Libramont; Eeckhout B, AZ St Dimpna, Geel; Eenkhoorn V, St Jozef Kliniek, Bornem; Emsens L, AZ OLV Ter Linden, Knokke-Heist; Fery F, Hôpital Erasme, ULB; Bruxelles; France A, UZA, Edegem; Gerard J, Private, Plainevaux; Ghys C, UZ Brussel, Brussel (Jette); Gies I, UZ Brussel, Brussel (Jette); Gillard P, UZ Leuven Gasthuisberg, Leuven; Herbaut C, CHU Brugmann, Bruxelles; Heyns E, AZ Groeninge Campus OLV, Kortrijk; Hilbrands R, UZ Brussel, Brussel (Jette); Joosen P, Maria Ziekenhuis Noord Limburg, Overpelt; Jopart P, Hôpital de Jolimont, Haine-St-Paul; Keymeulen B, UZ Brussel, Brussel (Jette); Kleynen P, CHU Saint-Pierre, Bruxelles; Kockaerts Y, ZOL Campus A. Dumont, Genk; Laga K, St Franciscus Ziekenhuis, Heusden Zolder; Lapauw B, UZ Gent, Gent; Lebrethon M, CHR de la Citadelle, Liège; Leus J, AZ Maria Middelares, Gent; Logghe K, H Hart Ziekenhuis, Roeselare; Maes T, Imeldaziekenhuis, Bonheiden; Martens M, AZ Sint-Jozef, Turnhout; Massa G, Jessa Ziekenhuis - Campus Virga-Jesse, Hasselt; Mathieu C, UZ Leuven Gasthuisberg, Leuven; Mekeirele K, OLV van Lourdes, Waregem; Messaaoui A, HUDERF, Bruxelles; Monballyu J, AZ KLINA, Brasschaat; Moorkens G, UZA, Edegem; Mortelmans K, Regionaal Ziekenhuis Heilig Hart, Leuven; Mortzos N, Jessa Ziekenhuis - Campus Virga-Jesse, Hasselt; Mouraux T, UCL Mt. Godinne, Yvoir; Mullens A, Jessa Ziekenhuis - Campus Virga-Jesse, Hasselt; Nobels F, OLV Ziekenhuis, Aalst; Nollet A, Regionaal Ziekenhuis Jan Yperman, Ieper; Ooms V, St Augustinus, Wilrijk; Paquot N, CHU Sart Tilman, Liège; Parent A, CHU Sart Tilman, Liège; Peiffer F, UZA, Edegem; Philips J, CHU Sart Tilman, Liège; Poschet K, St Vincentius Ziekenhuis, Antwerpen; Radermecker R, CHU Sart Tilman, Liège; Robbrecht S, AZ St Blasius, Dendermonde; Rocour-Brumioul D, CHR de la Citadelle, Liège; Ruige J, AZ Nikolaas, Sint-Niklaas; Scarnière D, Hôpital St Joseph, Gilly; Seret N, CH St Joseph, Liège; Sirault B, CHU Charleroi, Charleroi; Spincemaille K, H Hart Ziekenhuis, Roeselare; Strivay M, CHR de la Citadelle, Liège; Taelman P, AZ Maria Middelares, Gent; Taes Y, AZ St Jan, Brugge; Tenoutasse S, HUDERF, Bruxelles; T'sjoen G, UZ Gent, Gent; Tuyttens C, AZ St Lucas, Gent; Twickler M, AZ Monica, Antwerpen; Van Acker K, Centre de santé des Fagnes Clinique Chimay, Chimay; Van Aken E, AZ Diest Campus Statiestraat, Diest; Van Aken S, UZ Gent, Gent; Van Crombrugge P, OLV Ziekenhuis, Aalst; Van Den Bruel A, AZ St Jan, Brugge; Leuven Gasthuisberg, Leuven; Van Doorn J, H Hart Ziekenhuis, Lier; Van Huffel L, OLV Ziekenhuis, Aalst; Van Imschoot S, AZ St Jan, Brugge; Van Pottelbergh I, OLV Ziekenhuis, Aalst; Van Rooy P, ZNA Middelheim, Antwerpen; Vanbesien J, UZ Brussel, Brussel (Jette); Vandemeulebroucke E, AZ Jan Portaels, Vilvoorde; Vandenbroucke M, AZ Heilige Familie, Reet; Vanderstappen H, St Franciscus Ziekenhuis, Heusden Zolder; Vanfleteren E, St Jozefskliniek, Izegem; Vanhaverbeke G, AZ Groeninge Campus OLV, Kortrijk; Vercammen C, Imelda Ziekenhuis, Bonheiden; Verhaegen A, Jan Palfijn Ziekenhuis, Merksem; Verjans V, AZ St Jozef, Turnhout; Verniest R, AZ KLINA, Brasschaat; Vets B, Imelda Ziekenhuis, Bonheiden; Vieillevoye G, Clinique Notre Dame, Charleroi; Vinck W, St Augustinus, Wilrijk; Vinken S, Algemeen Stedelijk Ziekenhuis, Aalst; Weber E, Clinique St Joseph, Arlon.
